# Maternal visceral adipose tissue during the first half of pregnancy predicts gestational diabetes at the time of delivery – a cohort study

**DOI:** 10.1371/journal.pone.0232155

**Published:** 2020-04-30

**Authors:** Alexandre da Silva Rocha, Juliana Rombaldi Bernardi, Salete Matos, Daniela Cortés Kretzer, Alice Carvalhal Schöffel, Marcelo Zubaran Goldani, José Antônio de Azevedo Magalhães

**Affiliations:** 1 Graduate Program in Gynecology and Obstetrics, Universidade Federal do Rio Grande do Sul, Porto Alegre, Brazil; 2 Department of Nutrition, Graduate program in Child and Adolescent Health and Graduate Program in Food, Nutrition and Health, Hospital de Clínicas de Porto Alegre, School of Medicine, Universidade Federal do Rio Grande do Sul, Porto Alegre, Brazil; 3 Graduate Program in Child and Adolescent Health, Universidade Federal do Rio Grande do Sul, Porto Alegre, Brazil; 4 Department of Social and Behavioural Health Sciences, Dalla Lana School of Public Health, University of Toronto, Toronto, Canada; 5 Department of Pediatrics, Hospital de Clínicas de Porto Alegre, School of Medicine, Universidade Federal do Rio Grande do Sul, Porto Alegre, Brazil; 6 Maternal-Fetal Division (Head), Hospital de Clínicas de Porto Alegre, School of Medicine, Universidade Federal do Rio Grande do Sul, Porto Alegre, Brazil; University of Cambridge, UNITED KINGDOM

## Abstract

**Background:**

Gestational diabetes mellitus (GDM) is a common condition, often associated with high maternal and fetal morbidity. The use of new tools for early GDM screening can contribute to metabolic control to reduce maternal and fetal risk. This study aimed to ascertain whether maternal visceral adipose tissue (VAT) measurement by ultrasound during the first half of pregnancy can predict the occurrence of GDM during the third trimester.

**Methods:**

A prospective cohort study of 133 pregnant women with gestational age ≤20 weeks in an outpatient setting. VAT depth was measured by ultrasound at the maternal periumbilical region. GDM status was obtained through hospital charts during hospitalization to delivery. A Receiver Operator Characteristic (ROC) curve was used to determine the optimum threshold to predict GDM.

**Results:**

According to the ROC curve, a 45mm threshold was identified as the best cut-off value, with 66% of accuracy to predict GDM. Crude and adjusted odds ratios (OR) for GDM were 13.4 (95%CI 2.9–61.1) and 8.9 (95%CI 1.9–42.2), respectively. A similar result was obtained among pre-gravid non-obese women, with crude and adjusted OR of 16.6 (95%CI 1.9–142.6) and 14.4 (95%CI 1.7–125.7), respectively. Among pre-gravid obese patients, a 45mm threshold did not reach statistical significance to predict GDM.

**Conclusion:**

The high and significant OR found before and after adjustments provides additional evidence of a strong association between VAT and GDM. It appears that VAT measurement during the first half of pregnancy has great potential in identifying non-obese women at high risk for GDM. This evidence can assist obstetricians in correctly allocating resources among populations of pregnant women at risk, determined not only by pre-gravid body mass index (BMI).

## 1. Introduction

Gestational diabetes mellitus (GDM) is a common condition, often associated with high maternal and neonatal morbidity [1,2]. The prevalence of GDM may range from 2% to 40% according to population, diagnostic criteria, and geographic location. In addition, this prevalence is increasing, secondary to rates of obesity, ethnic diversity, more advanced maternal age, and recent changes in diagnostic criteria [3]. Treatment of GDM effectively prevents macrosomia, shoulder dystocia, and hypertensive disorders of pregnancy, including pre-eclampsia [4]. There is adequate evidence regarding the effectiveness of GDM treatment at reducing the risk of adverse outcomes through a low number needed to treat [1]. Approximately 70% to 80% of pregnant women diagnosed with GDM will achieve control of blood glucose levels with lifestyle changes alone (e.g., dietary reeducation and moderate physical exercise) [5]. The pathophysiology of GDM is closely related an imbalance as a result of peripheral insulin resistance that leads to increased amounts of circulating glucose. Pregnancy naturally leads to an increase in insulin secretion from the pancreas, stimulated by lactogen and prolactin. On the other hand, placental secretion of diabetic hormones such as growth hormone, corticotropin-releasing hormone, placental lactogen and progesterone leads to increased peripheral insulin resistance. When this balance tends to maintain high and prolonged maternal glucose levels, the condition of gestational diabetes mellitus will be present. [8]

GDM is diagnosed through altered fasting glucose or abnormal results on a glucose tolerance test (GTT) or glucose challenge test (GCT), usually performed during the second trimester. However, the blood glucose cutoff levels used to diagnose GDM were arbitrarily designated as those that increase the risk of diabetic fetopathy (macrosomia, excess fetal adiposity, fetal hyperinsulinemia) by a 1.75-fold. In addition, during the first trimester and the early second trimester, before glucose tolerance testing is usually performed, pre-existing type 2 diabetes mellitus and early GDM can lead to prolonged postprandial hyperglycemia, increasing metabolic risk to the mother and neonate [6].

The use of new tools for early GDM screening that combines safety, low cost and high efficacy can contribute to metabolic control as a method of reducing maternal and fetal risk. Visceral fat is known to be strongly associated with disease risk, especially those related to increased insulin resistance, heart disease and hypertension. The pathophysiology of central fat-related diseases involves three mechanisms: 1) the release of large amounts of free fatty acid in the liver, initiating decompensation with hyperinsulinemia, dyslipidemia and hyperglycemia; 2) visceral adipocytes capable of releasing visfatin, a cytokine related to diabetes and cardiovascular disease; and 3) visceral adiposity associated with the hypothalamus-pituitary-adrenal axis, leading to sympathetic overactivity [7]. Maternal visceral fat composition, as quantified by ultrasonography, has been associated with altered fasting glucose [8] [9], altered glucose tolerance test [10], pre-eclampsia [11], and preterm delivery [11]. Nevertheless, all of these studies evaluated non-obese, overweight and obese patients as a single group. Among adults, it is well known that central adiposity is a risk factor for metabolic syndrome and cardiovascular disease, even among non-obese men and women [12]. However, it is unknown if VAT can identify non-obese and overweight pregnant individuals at an increased risk and obese pregnant individuals at a reduced risk for GDM.

Within this context, the main aim of this study is to ascertain whether VAT measured by ultrasound during the first half of pregnancy can predict the presence of GDM at the time of delivery among obese, overweight and non-obese patients.

## 2. Methods

### 2.1 Sample

This study sample consisted of a prospective cohort of 154 pregnant women. Participants were approached from October 2016 to December 2017 at the Ultrasound Department of the Murialdo Teaching Health Center, a clinic that provides fetal medicine to the Public Health System in Porto Alegre City, Brazil. Participants were reevaluated during hospitalization for childbirth throughout five hospitals of the city's Public Health System. The reevaluation consisted of obtaining information regarding conditions during birth and, if applicable a diabetes diagnosis from the hospital medical records. Of the 154 women selected initially, 21 (13%) were lost to follow-up, resulting in a final sample of 133 women. The inclusion criteria were pregnant individuals with a gestational age of ≤20 weeks. The exclusion criteria were pregnancies with known fetal malformations or aneuploidy, scar tissue in the abdominal site that would preclude proper ultrasound visualization of VAT, and preexisting type 1 or 2 diabetes mellitus. All women who agreed to participate provided written informed consent.

### 2.2 Procedure

A routine obstetric ultrasound was performed by the author ASR (certified sonologist by the Brazilian College of Radiology) for diagnosis of fetal growth as described by Hadlock et al. [13]. The result was compared with fetal age estimated from the last menstrual period or previous ultrasound. Position of the placenta, fetal heartbeats, estimated amniotic fluid level, and basic fetal anatomy were assessed in all cases. Initially pregnancies were evaluated by endovaginal ultrasound for gestational age diagnosis, the position of the placenta, embryonic or fetal heart beats, yolk sac measurement, and the examination of maternal adnexa. In cases with a gestational age of 11+0 to 13+6 weeks, nuchal translucency and the nasal bone were evaluated aiming the detection of aneuploidy as described by Nicolaides et al. [14].

#### Maternal Visceral Adipose Tissue (VAT) measurement

**Fig 1:**
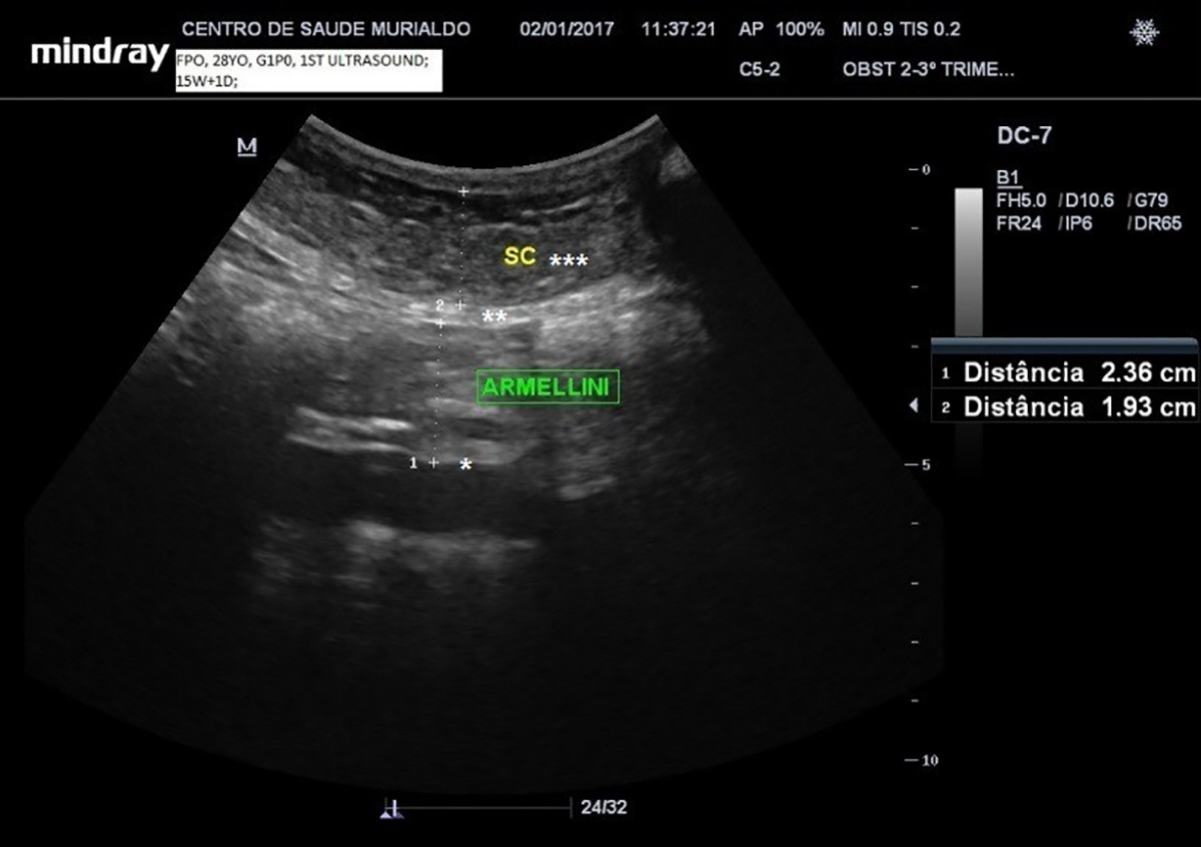
VAT ultrasound measurement according to the *Armellini´s* technique. (*) Abdominal aortic anterior wall. (**) *Linea* Alba. (***)Subcutaneous fat.

VAT was assessed with ultrasound electronic calipers, placed from the aortic anterior wall to the *linea* alba, located 2 cm above the maternal umbilical scar with the probe in the sagittal position as described by *Armellini* et al. [15] (Fig 1). The mean of two measurements, one obtained during maternal inspiration and one during expiration, was used to estimate VAT.

#### Anthropometry

The first measurement of maternal weight before the 12th week of pregnancy and the maternal height measured at the time of study inclusion were used to calculate the pre-gravid BMI. If weight data before 12 weeks of gestation were missing, pre-gravid maternal weight records were used to calculate the pre-gravid BMI.

Demographic and laboratory data: Routine outpatient antenatal records were used to assess the characteristics of fasting glucose, TTG or GCT during the sampling inclusion process. Demographic variables (i.e. age, ethnicity, tobacco use among others) were also extracted from antenatal records.

#### Gestational outcomes

Hospital records were assessed to, GDM treatment, GTT or GCT results together with newborn weight, Cesarean section (C-section) or vaginal birth and other maternal, fetal and neonatal conditions. The authors stated GDM positive as an abnormal GTT or GCT result or a fasting blood glucose level above the threshold outlined by the International Association of Diabetes and Pregnancy Study Groups (IADSPG). In the absence of records regarding diabetic diagnostic tests, the authors considered the identification of GDM in medical records during hospitalization as positive result.

### 2.3 Statistical analysis

Statistical analyses were performed in SPSS (Statistical Package for the Social Sciences) 23.0 for Windows. The level of significance was set at p < 0.05, with 95% confidence intervals for the odds ratio (OR) estimation.

On bivariate analysis, Student’s *t-*test and the *Mann–Whitney* test were used to compare means and medians between two groups. *Pearson’s* chi-square test was used to analyze the categorical variables. Sample characteristics were stratified by pre-gravid non-obese (BMI<30 kg/m^2^) versus obese (BMI≥30 kg/m^2^) status. A ROC curve was used to identify the best cutoff VAT measurement to predict GDM among all patients. Binary logistic regression was performed to estimate OR for GDM among different VAT measurements. In addition, subgroup analyses were performed for obese and non-obese patients. Adjusted ORs for GDM were estimated for potential confounders, such as maternal age and pre-gravid BMI, through binary logistic regression.

The study was submitted to the Research Ethics Committee of the municipality of Porto Alegre and received general approval number 2.132.090.

## 3. Results

The total sample (n = 133) was divided according to the pre-gravid BMI (Table 1). The mean age was 26 (± 6.2) years. Most participants were Caucasian, with a past of two pregnancies, and enrolled approximately during the 15^th^ week of pregnancy. Fasting glucose evaluated at enrollment was different between groups (79.6 mg/dL among non-obese versus 83.9 mg/dL among obese women). Need for C-section also differed between groups; approximately one-third of obese women versus only 18% of non-obese women required the surgical procedure. Birth weight was the same between groups at approximately 3.2 kg. Among the 133 patients included, the authors found 18 diagnoses of gestational diabetes, an incidence of 13.5%. Among cases of gestational diabetes, the presence of pre-gravid obesity (BMI ≥30.1 kg/m^2^) was found in 55.6%.

**Table 1:** Sample characteristics.

Variable	Pre–pregnancy BMI** < 25.0 kg/m^2^ (n = 53)	Pre-pregnancy BMI 25.0–30.0 kg/m^2^ (n = 36)	Pre–pregnancy BMI ≥ 30 kg/m^2^(n = 44)	P–*Value*
Age (yo)	25.6±6.1	27.7±6.9	27.0 ± 6.2	0.24
Non-Caucasian	49.1% (26)	47.2% (17)	34.1% (15)	0.341
Past Pregnancies^$^	2 (1–2.25)^a^	1 (1–3)^b^	2 (2–4)^a^	**<0.05**
Gestational age at inclusion (weeks)	15.8±4.0	14.9±3.9	15.4±3.6	0.603
Fasting glucose (mg/dl)	79.2±8.4^a^	80.7±8.4^a^^,^^b^	84.7±10.6^b^	**<0.05**
GDM	7.5% (4)	11.2% (4)	22.7% (10)	0.08
C-section	15.1% (8)	22.2% (8)	36.4% (16)	**<0.05**
VAT* (mm)	37.0±12.5^a^	44.0±11.2^b^	53.1 ±14.8^c^	<0.01
25 percentiles	28.3	36.3	40.3	
50 percentiles	36.7	42.9	50.4	
75 percentiles	46.3	53.0	62.9	
Mean newborn weight(g)	3.178±424	3.354±436	3.273 ±591	0.247

(*) Maternal visceral adipose tissue measured according *Armellini´s* technique

(^$^) Median (interquartile range, Kruskall Wallis test)

(**) Body Mass Iindex

(^a,b,c^) different letters are statistically different; GDM: gestational diabetes mellitus; VAT: maternal visceral adipose tissue.

VAT measured by the *Armellini´s* technique was significantly different between the non-obese (*m =* 4.0 cm) and obese (*m =* 5.3 cm) groups (p < 0.001). Regarding the diabetes outcome, the authors found significantly different VAT means between pregnant women with diabetes (VAT = 55.4 ±11.4mm) and non-diabetic (VAT = 42.5±11.4mm).

The ability of VAT to predict GDM was evaluated according to two thresholds: 45 mm, which was the optimal point of the ROC curve to discriminate high GDM risk in the sample as a whole; and 47 mm, the cutoff defined in previous studies examining VAT in patients with ]abnormal GCT [16] and abnormal GTT [8].

In the sample of 133 pregnant women (Table 2), the 45mm threshold was better to discriminate GDM risk, with significant crude and adjusted odds ratios of 13.4 (95%CI 2.9–61.1) and 8.9 (95%CI 1.9–42.2) respectively. A similar result was obtained among pre-gravid non-obese women, with crude and adjusted odds ratios of 16.6 (95%CI 1.9–142.6) and 14.4 (95%CI 1.7–125.7) respectively. In pre-gravid obese women, neither the 45mm nor the 47mm VAT threshold was significantly able to predict GDM risk. The 45mm VAT threshold had 66% accuracy in predicting GDM in the sample as a whole and 72% among non-obese pre-gravid women.

**Table 2:** Performance of maternal visceral adipose tissue measurement and pre-pregnancy Body Mass Index (BMI) to predict gestational diabetes mellitus.

		Crude OR (CI 95%)	Adjusted OR (CI 95%)	Sens.	Spec.	PPV	PNV	Acu.
**Total sample**								
	VAT 47mm	6.8 (2.1–22.1)	3.8 (1.1–13.5)^#^	77.8	66.1	26.9	94.0	67.7
	VAT 45mm	13.4 (2.9–61.1)	8.9 (1.9–42.2)^#^	88.9	62.6	27.1	97.3	66.2
**Pre-gravid Obese**								
	VAT 47mm	7.0 (0.8–62.0)	5.8 (0.6–54.1)*	90.0	43.8	33.3	93.3	54.8
	VAT 45mm	7.1 (0.8–62.5)	6.1 (0.7–55.3)*	90.0	44.1	32.1	93.8	54.5
**Pre-gravid Non-obese**								
	VAT 47mm	5.0 (1.1–22.8)	4.0 (0.8–19.4)*	62.5	75.0	20.0	95.2	73.9
	VAT 45mm	16.6 (1.9–142.6)	14.4 (1.7–125.7)*	87.5	70.4	22.5	98.3	71.9
**Pre-gravid BMI**^**$**^ **> 30.1 kg/m**^**2**^		3.0 (1.1–8.2)	2.7 (0.96–7.5)*	55.6	70.4	22.7	91.0	68.4

(*) adjusted for maternal age

(^#^) adjusted for maternal age and pre pregnant BMI

OR: Odds Ratio; CI: Confidence Interval; VAT: maternal visceral adipose tissue; Sens: sensitivity; Spec: specificity; PPV: predictive positive value; PNV: predictive negative value; Acu: accuracy;

(^$^) BMI: body mass index.

## 4. Discussion

The main finding of this study is that ultrasound measurement of VAT during the first half of pregnancy can predict the risk of GDM during the third trimester, including in patients without classical metabolic risk factor such as pre-gravid obesity. In this group of patients, GDM is rarely diagnosed or even suspected until the second trimester, which may result in prolonged and elevated levels of maternal blood glucose in early pregnancy (especially in the postprandial period).

Previous studies [7,10,17–19] have assessed the use of ultrasound aiming GDM early screening, but, to our knowledge, this is the first to focus on non-obese patients, in a low-risk outpatient environment, and compare VAT findings with gestational outcomes.

The use of a 45mm VAT threshold to discriminate high versus low risk of GDM appeared to provide high sensitivity and specificity in this study, including in women with non-obese pre-gravid BMI (a group commonly believed to be at low risk of GDM). Furthermore, the high, significant odds ratio found before and after adjustments provides additional evidence of a strong association between VAT and GDM. On the other hand, obese pregnant women with a VAT<45mm showed no significant tendency toward protection against GDM, with a high negative predictive value. We believe that a larger sample of pre-gravid obese women with VAT<45mm would strengthen a protective effect against GDM late in pregnancy. These findings could help obstetricians correctly allocate resources in populations of pregnant women at metabolic risk, identified not only by pre-gravid BMI.

Our study is consistent with previous reports of an association between abnormally high visceral adiposity and GDM risk. Martin et al. found VAT>47mm was significantly associated with abnormal second trimester GCT in a sample of 62 pregnant women, with an OR of 17.3 (95%CI 18–163.8) before adjustment and 16.9 (95%CI 1.5–194.6) after adjusting for maternal age and pre-gravid BMI [16]. A similar approach using a VAT threshold of 48mm found a significant association with abnormal second trimester 75-g GTT in 485 pregnant women [8]. Another recent study found that a 42.7mm VAT threshold could predict the risk of abnormal response to a 75-g GTT with 87% sensitivity (95%CI 60–98%) and 62% specificity in the early third trimester [20]. In previous studies, VAT measured during the nuchal translucency assessment also correlated significantly with fasting glucose, insulinemia, and insulin sensitivity (HOMA-IR Homeostasis Model Assessment) [7,21].

A Canadian study utilized a different statistical approach in a large sample (n = 1048) to compare different areas under the ROC curve among women stratified by pre-gravid BMI, gestational age, and VAT measured during the nuchal translucency ultrasound. The objective was predict abnormal GCT at 24–28 weeks of pregnancy. The authors found no differences in area under the ROC curve to predict GDM risk among the three scenarios. However, the inclusion of VAT improved the detection rate to 43% in combination with gestational age and pre-gravid BMI. The authors highlighted the low prevalence of GDM in the sample (5.8%) as a possible reason for the poor performance of ultrasound [10]. A similarly low VAT predictive value was found in a Brazilian study among pregnant women included between 15 to 20 weeks, which compared VAT with fasting HOMA-IR, fasting insulin, and lipids measured during the third trimester. The authors concluded that VAT measured in the first half of pregnancy is not superior to pre-gravid BMI in predicting insulin resistance and related biochemical measures in later pregnancy. They propose three limitations that might explain poor ultrasound performance: late inclusion (around the 19^th^ week of pregnancy), technical aspects of ultrasound measurement (such as maternal body habitus), and, the inability of the authors to exclude women with metabolic syndrome or pre-existing GDM [17].

It has been extensively demonstrated that a BMI>35kg/m^2^ is associated with a fivefold increase in the risk of developing GDM [22]. However, the prognostic value of BMI is limited by the fact that it does not distinguish between excess fat, muscle or bone mass, nor does it reveal the distribution of adipose tissue. VAT may reflect cardiometabolic risk better than BMI [23]. This study suggests that the use of VAT measurement is better than pre-pregnancy BMI to predict GDM. First, ultrasound measurement has a much higher sensitivity for GDM than pre-pregnancy BMI (89% and 55%). Sensitivity is a core feature of a screening test. Second, pre-pregnant women with non-obese BMI who are at risk for GDM can be detected using VAT above 45mm with a sensitivity of 88%. Third, among obese women, a VAT<45 mm can suggest a group with low risk of GDM, with a negative predictive value of 94%. Finally, the use of the ultrasound screening method was superior to the pre-gravid BMI, with similar accuracy and specificity, but higher sensitivity, as well as positive and negative predictive values.

Visceral fat is formed by the greater and lesser *omentum* and mesenteric fat, which differs from the fat present in non-visceral regions. The main differences between the two adipose tissues include their endocrine function, lipolytic responses to insulin and other hormones. Regarding visceral fat, the anatomical position and the capability of draining the contents directly to the liver through the portal venous system impacts the hepatic inflow of free fatty acids and adipokines. This process leads to the hepatic production of inflammatory mediators, such as C-reactive protein, and others that contribute to the metabolic risk of pregnant women. This mechanism does not occur in adipose tissue located in non-visceral regions.

Regarding diabetes, VAT in non-pregnant adults is associated with 1) insulin resistance, which prevents glucose and additional fat from entering the cell and becoming preferentially oxidized; 2) glucose intolerance, with higher rates of insulin-stimulated glucose uptake compared with subcutaneous adipocytes; and 3) dyslipidemia, with greater lipolytic activity than subcutaneous adipocytes [24].

Several limitations of our study must be mentioned. First, ultrasound imaging was performed by a single fetal-imaging specialist, rather than by two examiners. The small sample of obese pregnant women limited the statistical power of VAT to predict the risk of GDM in this group. Information on the treatment used by pregnant women with GDM was unavailable due to poor record-keeping among hospital admission charts. Finally, the 13% attrition rate may have influenced some findings.

In conclusion, the use of a 45mm threshold of VAT to predict the risk of GDM seems to be a fast and inexpensive approach that can be used during the first 20 weeks of pregnancy, with the potential to improve early risk stratification, especially in pre-gravid non-obese women. For obese women, additional studies are needed to determine the best VAT cutoff to ensure low GDM risk later, thus improving resources allocation for referral to high-risk pregnancy care.
